# Context-Dependent Allelopathic Interactions Between Winter Cereal Rye and Grain Legumes in Relay-Row Intercropping Systems

**DOI:** 10.3390/biology15130983

**Published:** 2026-06-23

**Authors:** Lina Šarūnaitė, Rokas Antanynas, Aušra Arlauskienė, Andrew J. Price

**Affiliations:** 1 Institute of Agriculture, Lithuanian Research Centre for Agriculture and Forestry, LT-58344 Akademija, Lithuania; rokas.antanynas@lammc.lt (R.A.); ausra.arlauskiene@lammc.lt (A.A.); 2 USDA-ARS National Soil Dynamics Laboratory, 411 S. Donahue Dr., Auburn, AL 36832, USA; andrew.price@usda.gov

**Keywords:** plant competition, secondary metabolites, land equivalent ratio, organic farming, agroecology, species interactions

## Abstract

Plant–plant interactions in agroecosystems are shaped by both biochemical interference and resource-mediated competition, yet the relative contribution of these mechanisms under field conditions remains unclear. This study evaluated whether allelopathic activity expressed by winter cereal rye (*Secale cereale* L.) and grain legumes under controlled conditions is associated with competitive outcomes in relay-row intercropping under organic farming. Greenhouse bioassays and laboratory screening assays were combined with a field experiment conducted under certified organic farming conditions. Aqueous leachates and biomass extracts influenced early root development of a sensitive recipient species in a time- and concentration-dependent manner, indicating measurable biological activity. Secondary metabolite profiles differed significantly among species, particularly in belowground biomass. However, under field conditions, no persistent suppression of grain legumes was observed. Aboveground biomass accumulation and land equivalent ratio (LER) values exceeded 1.0 in all intercrops, with the highest LER recorded for the cereal rye–lentil combination. The results suggest that under the conditions of this study, resource partitioning, species compatibility, and other ecological interactions may have had a greater influence on intercrop performance than the allelopathic effects detected under controlled conditions.

## Simple Summary

Intercropping cereals and legumes is widely used in sustainable and organic farming to improve resource use and crop productivity. Plant interactions in these systems may be influenced by both competition for resources and allelopathy, a process in which plants release compounds that affect the growth of neighboring plants. In this study, we investigated whether the allelopathic activity of winter cereal rye and grain legumes observed under controlled laboratory and greenhouse conditions was reflected in their performance when grown together under organic field conditions. Although biological activity was detected in laboratory assays and differences in secondary metabolites were identified among species, these effects did not result in long-term suppression of legumes in the field. All intercrops produced land equivalent ratio values above 1.0, indicating more efficient use of resources compared with monocultures. The findings suggest that resource sharing, species compatibility, and other ecological interactions play a more important role in determining intercrop performance than allelopathic effects alone.

## 1. Introduction

Agricultural systems across Europe and North America face increasing pressure to reconcile productivity with environmental sustainability, as intensive monoculture-based production has contributed to biodiversity decline and ecosystem degradation [1]. Traditional cropping systems often rely heavily on mineral fertilizers and pesticides, weakening ecological regulation processes and increasing environmental risks.

Diversified cropping strategies are increasingly considered a key pathway toward improving agroecosystem resilience and soil health restoration; thus, discussions concerning crop diversification and its relationship to “regenerative agriculture” are increasing [2]. Intercropping, defined as the cultivation of two or more crop species in spatial and temporal proximity, enhances functional biodiversity and can improve nutrient use efficiency, soil fertility, yield stability, and weed suppression [3,4,5]. Recent metadata syntheses emphasize that intercrop performance is governed by complex interactions among resource partitioning, spatial configuration, plant functional traits, and belowground processes [6]. Therefore, optimizing intercropping systems requires mechanistic understanding of plant–plant interactions rather than relying solely on empirical yield comparisons.

Cereal–legume mixtures are among the most widely adopted intercrops in Europe and North America due to their agronomic and ecological benefits [7,8,9]. Winter cereal rye (*Secale cereale* L.) is particularly valued in more northern-latitude organic agroecosystems for its rapid establishment, competitiveness, cold tolerance, weed suppression capacity, and ability to improve soil structure [10,11]. Grain legumes, including pea, lentil, and chickpea, contribute protein-rich grain and biologically fixed nitrogen, supporting soil fertility and system sustainability. These species differ in growth habit, competitiveness, rooting patterns and adaptation to environmental conditions, making them suitable models for evaluating species-specific interactions in relay-row intercropping systems.

However, intercrop performance depends on the balance between complementarity and competition between crops. Differences in rooting depth, growth dynamics, canopy architecture, and nutrient acquisition strategies may enhance resource use efficiency or lead to competitive imbalance [12]. Understanding the mechanisms that regulate these interactions remains central to improving intercropping design and adoption.

Within this mechanistic framework, allelopathy represents one ecological process that may contribute to plant–plant interactions. Allelopathic effects arise from the release of secondary metabolites through roots or decomposing residues, potentially influencing seed germination and early seedling growth [13,14]. Early plant developmental stages are particularly sensitive to allelochemicals, which may interfere with physiological processes such as nutrient uptake, photosynthesis, and cell division. Nevertheless, under field conditions, allelopathic effects operate alongside resource competition, spatial crop arrangement, root system architecture, mycorrhizal processes, and soil microbial-mediated transformations [13,15]. Consequently, their ecological relevance is strongly context-dependent.

Although allelopathic activity has been widely demonstrated under controlled laboratory conditions [16,17], its functional role within field-scale intercropping systems remains insufficiently understood. Plant-derived allelochemicals may act directly through biomass leachate or indirectly via soil-mediated pathways, and their expression can vary across species combinations and environmental conditions [18]. Integrative studies linking controlled bioassays with field experiments are therefore necessary to clarify how biochemical potential interacts with ecological processes in agroecosystems.

In this study, interactions between cereal rye and legume species were evaluated in two complementary experiments: (1) greenhouse bioassays to quantify measurable allelopathic activity under controlled conditions; and (2) a relay-row intercropping field experiment under organic farming conditions to determine how this biochemical potential interacts with resource competition and species-specific traits. Winter cereal rye was selected due to its intercontinental agronomic adoption, growth traits, and potential role as a living mulch in cereal–legume systems. The main objective was to evaluate the competitive relationships and ecological compatibility of winter rye with different grain legumes in a relay intercropping system.

This study integrates controlled allelopathic bioassays with field-scale relay intercropping to evaluate whether biochemical potential translates into agronomic effects under organic conditions. Unlike most previous studies, we directly compare controlled and field responses to assess the ecological relevance of allelopathy.

We hypothesize that cereal rye and legume species exudates result in measurable allelopathic activity under controlled conditions but that under field conditions this biochemical potential is moderated by resource competition and species traits, shaping competitive outcomes reflected in biomass and yield in relay intercropping systems.

## 2. Materials and Methods

Controlled greenhouse experiment for evaluating potential allelopathic effects of two cover crop species. A greenhouse pot experiment was conducted at the USDA-ARS National Soil Dynamics Laboratory, Auburn, AL, USA, using a completely randomized design (CRD) with three treatments: The greenhouse experiment included three treatments arranged in a completely randomized design: (i) a control consisting of pots containing substrate only, without plants; (ii) winter cereal rye, selected due to its well-documented allelopathic potential associated with the production of benzoxazinoid compounds and their release from plant residues and other attributes previously mentioned [19,20]; and (iii) a legume, crimson clover (*Trifolium incarnatum* L.). Crimson clover was chosen as a representative legume cover crop model for preliminary screening, based on its rapid early growth and widespread use in cover cropping systems, as well as the documented allelopathic potential of leguminous plants and their residues [21]. Each treatment was replicated three times (n = 3). While crimson clover served as a model legume species under controlled conditions, the field experiment evaluated cereal rye in relay-row intercropping with cash grain legumes (pea, lentil, and chickpea) under certified organic farming conditions.

Pots (7.6 L) were filled with a homogeneous sphagnum peat-based general-purpose growing medium (PRO-MIX Bx; Premier Horticulture Inc., Quakertown, PA, USA) with a pH of 5.5–6.2 in the greenhouse for each treatment and arranged randomly on greenhouse benches. Greenhouse conditions were maintained at a 26/21 °C day/night temperature regime, with natural daylight. All pots were managed uniformly and rotated randomly throughout the experiment.

Plant cultivation and phenological stage selection. Cereal rye and crimson clover were grown for approximately eight weeks. Rye plants were grown until the early heading stage, while crimson clover plants were grown until the visible bud formation stage. These phenological stages were selected based on previous evidence that the synthesis and accumulation of allelochemical compounds increase during the transition from vegetative to reproductive development. During the growing period, plants were irrigated regularly to avoid water stress and ensure uniform growth. No nutrient limitations were imposed, and all treatments received identical management.

Plant termination and residue decomposition. After approximately eight weeks of growth, all plants were uniformly terminated by applying glyphosate (RoundupPowerMAX^®^, Bayer Crop Science, Monheim am Rhein, Germany) at 1.68 kg ae ha^−1^. The application was applied with a CO_2_-pressurized backpack sprayer equipped with TTI 11,004 nozzles (TeeJet^®^ Technologies, Glendale Heights, IL, USA) at 276 kPa calibrated to deliver 280 L ha^−1^ Following termination, the dead aboveground biomass was left intact on the substrate surface in each pot and was not removed throughout the decomposition period. This approach was used to allow the release of water-soluble allelochemicals from decomposing residues into the substrate, as is commonly applied in residue-mediated allelopathy studies [17,22].

Irrigation regime and leachate formation. Pots were irrigated to maintain consistent moisture conditions across all treatments. During each irrigation event, water was applied until leachate was first observed at the bottom of the pots. This procedure ensured uniform moisture distribution throughout the substrate profile and promoted the downward movement of water-soluble compounds released during residue decomposition, as commonly described in residue-mediated allelopathy studies [17,23].

After glyphosate application, irrigation frequency was adjusted according to the experimental design: pots were watered at 3-day (3 DAP), 9-day (9 DAP), and 15-day (15 DAP) intervals. Irrigation continued throughout the decomposition period to support controlled biomass breakdown and the potential release of allelochemicals into the substrate.

Field experimental design. The field experiment was conducted in 2023 at the Lithuanian Research Centre for Agriculture and Forestry (LAMMC), Akademija, Central Lithuania (55°24′ N, 23°51′ E). The trial was established under certified organic farming conditions to evaluate intercropping systems based on greenhouse experiment results. In accordance with the management practices of the experimental organic field, no fertilizers, irrigation, or weed, pest, and disease control measures were applied during the study period. The soil at the experimental site was an *Endocalcari–endohypogleyic Cambisol* (loam), with topsoil (0–25 cm) properties of pH 6.6–7.1, available P_2_O_5_ 81–121 mg kg^−1^, K_2_O 131–144 mg kg^−1^, and organic carbon 0.89–0.98%.

**Figure 1:**
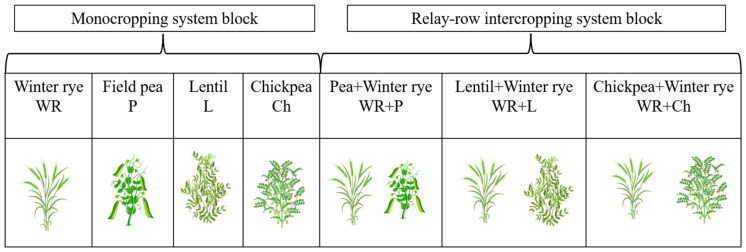
Field layout of different relay-intercropping systems with cereal rye and a spring grain legume. Note: WR + P and WR + Ch represent the layout of one cereal rye row (12.5 cm spacing between the rows of the cereal rye and grain legume; the total width of the cereal rye row strip was 25 cm), two grain legume rows (12.5 cm spacing between the rows of the grain legumes; the total width of the grain legume row strip was 50 cm).

The experiment was initiated in spring 2023 to assess relay-row intercropping systems of grain legumes with cereal rye (*Secale cereale* L., cv. ‘VB Duoniai’), where rye served as an inter-row cover crop/living mulch. Legume species were included: semi-leafless field pea (*Pisum sativum* L., cv. ‘Lina DS’), lentil (*Lens culinaris* Medik., cv. ‘Danaja’) and chickpea (*Cicer arietinum* L., cv. ‘Sokol’). Both species were grown as sole crops and in relay-row intercrops (Figure 1). All crops were sown on the same day (19 May 2023).

In the relay-row system, two rows of cereal rye alternated with one row of legumes seeded at the same sowing depth. The preceding crop at the experimental field site was winter wheat. Soil preparation consisted of autumn plowing followed by shallow spring cultivation. Sowing was performed using a conventional seed drill; the relay-row configuration was achieved by operating the drill in two passes with selected coulters closed.

Seeding rates in sole crops were 1.1 million seeds ha^−1^ for cereal rye, 1.1 million seeds ha^−1^ for field pea, 3.2 million seeds ha^−1^ for lentil and 0.8 million seeds ha^−1^ for chickpea. In relay-row intercrops, the same within-row seeding rates were used, while the overall rye-to-legume seed ratio was adjusted to 0.3:1 relative to monoculture density. The rye-to-legume seed ratio was selected to balance the ecological functions of rye as a living mulch with the productive role of grain legumes as the main crop. Reduced rye density was used to limit competition for light, water, and nutrients while ensuring adequate rye establishment and tillering.

Grain legumes were harvested at full maturity, and crop residues were chopped and evenly distributed. Cereal rye biomass was mulched twice during the growing season to promote tillering and allowed to regrow for harvest in the subsequent season. Plot size was 20 × 3 m (60 m^2^), with a harvested area of 39.6 m^2^. Treatments were arranged in a randomized complete block design with four replications.

Land equivalent ratio (LER). LER was calculated according to Mead and Willey [24], as the ratio of component biomass in intercropping to that in monocropping. For each crop component, LER was calculated as the ratio of its aboveground dry biomass in intercropping to its aboveground dry mass in monocropping:

$$LER_{rye}=\frac{Y_{rye,int}}{Y_{rye,mono}}$$

$$LER_{legume}=\frac{Y_{legume,int}}{Y_{legume,mono}}$$

where $Y_{rye,int}$ and $Y_{legume,int}$ represent the aboveground dry mass (t ha^−1^) of cereal rye and grain legume, respectively, measured in the intercropping system, while $Y_{rye,mono}$ and $Y_{legume,mono}$ denote the corresponding aboveground dry mass obtained in monocropping.

The total land equivalent ratio was calculated as the sum of the component LERs:

$$LER_{total}=LER_{rye}+LER_{legume}$$

A value of $LER_{total}>1$ indicates more efficient land use in intercropping compared with monocropping, whereas $LER_{total}=1$ indicates equal land-use efficiency.

Allelopathy bioassay. Allelopathic effects were evaluated using two complementary bioassays. Prior to the establishment of the field experiment, greenhouse and laboratory allelopathy bioassays were conducted utilizing a modified Pederson [25] procedure to screen the compatibility of cereal rye and legume species and to support the selection of crop combinations for field evaluation. Radish (*Raphanus sativus* L.) was utilized as an indicator species because of its relatively moderate to small seed size and uniform growth habit.

First, aqueous leachates were obtained from potting substrates without plants, with cereal rye (*Secale cereale* L.) and crimson clover (*Trifolium incarnatum* L.) grown under controlled greenhouse conditions. After plants reached the target phenological stages and were terminated, aboveground biomass was left on the substrate surface. Pots were irrigated until leachate appeared at the bottom and collected, following the release of water-soluble compounds during residue decomposition.

Second, aqueous extracts were prepared from fresh root biomass of field-grown cereal rye, field pea (*Pisum sativum* L.), and chickpea (*Cicer arietinum* L.), grown as sole crops and intercrops. Undamaged shoots were collected prior to flowering. Extracts were prepared by homogenizing 250 g of fresh tissue in 1 L of distilled water, incubated at 20 °C in darkness for 24 h, filtered, and stored at 4 °C until use [26].

In the greenhouse experiment, collected leachates were used undiluted at 100% concentration, with distilled water as the control. The leachate was centrifuged at 4000 revolutions per minute for 15 min. Next, the extract was filtered through Whatman No. 42 paper. The solution was stored in the refrigerator at 4 °C until use. Radish seeds were sterilized in 0.6% (*v*/*v*) sodium hypochlorite (NaOCl) for 10 min and rinsed thoroughly with running water. Seeds were allowed to pre-germinate on moist paper towels for 36 h. The nutrient agar (28 g L^−1^) was autoclaved at 121 °C for 15 min. Before mixing, the agar was cooled to 50 °C. Then, agar was mixed with cover crop extract in an equal ratio (1:1). The cover-agar extract solution was poured into disposable Petri dishes and allowed to solidify before placing the seed. Ten pre-germinated seeds having a radicle length of less than 2 mm were placed on each agar plate. Mixing, pouring of the agar-cover extract and seed placing were done under the laminar flow hood to create sterile conditions. The laminar flow hood was cleaned every time with ethanol. Plates were sealed with parafilm and kept in a growth chamber. The environment of the growth chamber includes temperature (25 °C during the day and 22 °C at night), a 14 h daylight period, and relative humidity of 65%. The experiment was conducted three times.

The radicle length (mm) of each seed was measured after 12, 24, and 48 h using a digital caliper. The length of all the seeds present in the Petri dish from each plate was averaged to give a plate value. Plate values from all three runs were combined into a dataset for each species. In contrast, extracts prepared from field-grown experimental plants for bioassays were diluted with distilled water to final concentrations of 25%, 50%, and 75%, with distilled water used as the control. Radish seeds exposed to field-derived extracts were as above, and germination parameters were recorded after 72 h.

Statistical analysis. Data was analyzed using one-way analysis of variance (ANOVA), with treatment as the main factor. Differences among treatment means were considered statistically significant at *p* < 0.05. Mean comparisons were performed using Duncan’s multiple range test. Because the greenhouse bioassays, biochemical analyses and field assessments represented separate experimental components with different objectives and response variables, statistical analyses were performed independently for each dataset. The objective was to compare treatment effects within individual experimental components and assessment periods rather than to develop a single multifactorial model integrating all factors simultaneously. All statistical analyses were conducted using SAS software, version 9.4 (SAS Institute Inc., Cary, NC, USA, 2002–2010).

## 3. Results

### 3.1. Polyphenolic Composition and Biological Activity of Cereal Rye and Legumes

#### 3.1.1. Allelopathic Potential of Cereal Rye and Legumes: Bioassay Results Under Controlled Conditions

The greenhouse bioassay demonstrated that radish root length responded to aqueous leachates depending on the time after plant termination and incubation period (Table 1). However, the observed effects were temporally limited and not clearly species-specific.

At 3d after plant termination (3 DAP), no statistically significant differences among treatments were detected at 12, 24, or 48 h after germination (Pr > F = 0.149–0.407), indicating that biological activity associated with early residue decomposition was either absent or below detectable levels.

At 9 DAP, significant treatment effects were observed at 24 and 48 h of incubation (Pr > F = 0.009 and 0.001, respectively), with the model explaining 35–45% of the variation (R^2^ = 0.354–0.454). Radish root length in the distilled water control was significantly greater than in all leachate treatments. However, no statistically significant differences were detected among substrate, crimson clover, and cereal rye leachates, as all belonged to the same statistical group. Although a consistent directional pattern was observed (control > substrate > clover > winter rye), the relatively high variability among replicates (SD up to 18.74 mm) combined with the limited number of replications (n = 3) restricted the statistical power to resolve interspecific differences.

At 15 DAP, no significant differences among treatments were detected at any incubation time (Pr > F = 0.274–0.479). Although mean root length values in leachate treatments remained numerically lower than in control, increased variability resulted in non-significant differences.

Overall, significant inhibition of radish root elongation was detected only at 9 DAP and only relative to the distilled water control. These results indicate a transient biological effect associated with residue decomposition while providing no statistical evidence for distinct allelopathic dominance of either cereal rye or crimson clover under the conditions of this assay.

#### 3.1.2. Polyphenolic Composition and Biological Activity of Cereal Rye and Legumes Evaluated Under Lithuanian Conditions

Secondary metabolites and bioassay response. Secondary metabolite analyses were conducted on field-grown plant material sampled at an early growth stage corresponding to the period reported in the literature as critical for allelochemical activity [19]. The content of secondary metabolites differed significantly among crop species in both aboveground and underground biomass (Table 2). In aboveground biomass, the total contents of polyphenols and flavonoids were higher, whereas polyphenolic acids (except in pea) were lower compared with the underground plant parts. The highest total polyphenol values were observed in chickpea, while the lowest were found in cereal rye and lentil. Flavonoid content in aboveground biomass was highest in pea and chickpea, whereas cereal rye exhibited the lowest values; however, the differences were not statistically significant. Total polyphenolic acid content did not differ significantly among species in aboveground biomass.

Differences in secondary metabolites among species in underground biomass were statistically significant for all analyzed compound groups (*p* ≤ 0.0001–0.0090). In underground biomass, chickpea exhibited markedly higher contents of total polyphenols and polyphenolic acids compared with the other species. Compared with cereal rye, these differences reached 8.2- and 27.4-fold, respectively. The highest total flavonoid content was detected in pea underground biomass and did not differ significantly from that of chickpea. The results indicate that, under Lithuanian conditions, the roots of grain legumes, particularly chickpea, may represent a more important source of bioactive compounds than the underground biomass of rye.

**Figure 2:**
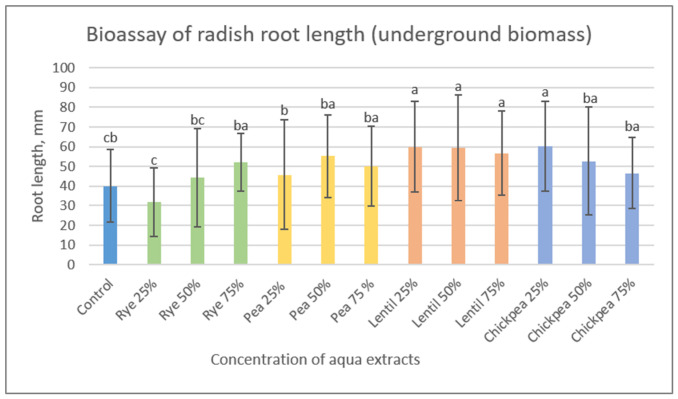
Radish root length following exposure to aqueous extracts derived from underground biomass of field-grown intercropping systems at increasing extract concentrations. Means followed by the same letters in the same column and section do not differ from one another (*p* < 0.05).

Radish root length varied depending on the type of aqueous extract and its concentration (Figure 2). Aqueous extracts derived from cereal rye underground biomass at 50% and 75% concentrations tended to stimulate radish root growth; however, these effects were not statistically significant. In contrast, the lower concentration (25%) of the cereal rye extract exhibited a slight inhibitory effect on root elongation.

The longest radish roots in response to pea biomass extract were observed at the 50% concentration, whereas increasing the concentration to 75% resulted in a 9.4% reduction in root length. Lentil biomass extract showed the strongest stimulatory effect on radish root growth regardless of extract concentration, with root length being 42.0–50.0% greater than that of the control. When chickpea biomass extract was applied, the longest radish roots were observed at the lowest concentration (25%), while increasing extract concentration led to a progressive reduction in root length.

The highest extract concentration (75%) reduced radish root growth (except for rye), indicating an inhibitory effect. Based on mean values across concentrations, plant extracts were ranked in increasing order of their effect on radish root growth as follows: cereal rye (42.76 mm) < pea (50.4 mm) < chickpea (53.17 mm) < lentil (58.80 mm). Compared with the control (approximately 40 mm), most grain legume extracts stimulated radish root growth, particularly those derived from lentil and chickpea. In contrast, rye extracts did not exhibit a clear growth-promoting effect, and the lowest extract concentration even reduced root length. Although statistically significant differences were observed among some treatments (*p* < 0.05), the relatively large standard deviation values indicate considerable variability in the results.

Based on these results, treatments showing the strongest and most consistent effects on radish root elongation were selected for subsequent field experiments.

### 3.2. Expression of Allelopathic Effects of Cereal Rye and Legumes Under Field Conditions in an Intercropping System

Early-season aboveground biomass accumulation. At five weeks after full emergence, biomass did not differ significantly among cereal rye treatments or among grain legume species, irrespective of the cropping system (Table 3). In most cases, cereal rye aboveground biomass increased, whereas grain legume biomass decreased compared with their respective monocrops. Aboveground biomass accumulation differed significantly among cropping systems during the second sampling period.

Compared with the 5-week biomass data, the greatest increase in cereal rye biomass after 8 weeks was observed in the WR+P and WR+L intercrops, with increases that were 1.5- and 2.2-fold higher, respectively, than in the cereal rye monocrop. In the WR+Ch intercrop, chickpea exhibited the highest biomass increase of 1783.2 kg ha^−1^ relative to the 5-week measurements; however, this increase was lower than that observed in the chickpea monocrop.

Over the 8-week period, biomass changes in the component crops in intercrops followed patterns similar to those observed at 5 weeks, but the differences were more pronounced. In intercrops, pea and lentil increased cereal rye biomass by 21.1–39.8% compared with the cereal rye monocrop, in contrast to chickpea. In all intercrops, grain legume biomass decreased. Pea and chickpea biomass was reduced by 41.8–49.9%, whereas lentil biomass remained largely unchanged relative to the corresponding monocrop.

Productivity and land equivalent ratio (LER). Field experiments revealed significant differences in total aboveground dry mass among cropping systems (Table 4). Five weeks after emergence, the WR and L monocrops produced significantly lower biomass yields than the other cropping systems. At eight weeks after emergence, the lowest biomass was recorded in the cereal rye monocrop, whereas all grain legume monocrops produced significantly higher biomass, with chickpea showing the highest values, followed by pea and lentil. Among intercrops, the cereal rye–lentil intercropping produced the highest biomass.

Total aboveground biomass in the WR+P and WR+L intercrops was 11.6% and 81.3% higher, respectively, than in the corresponding grain legume monocrops. In contrast, total biomass in the WR+Ch intercrop was reduced due to lower cereal rye and chickpea biomass, while the WR+P intercrop showed an intermediate response, with increased cereal rye but reduced pea biomass, resulting in total biomass similar to that of the pea monocrop.

In the WR+P and WR+L intercrops, grain legumes accounted for a slightly larger proportion of total aboveground biomass than cereal rye. In contrast, chickpea dominated the cereal rye–chickpea intercrop, accounting for 73.0% of the total aboveground biomass.

LER values calculated for individual crop components and intercrops varied among treatments (Table 4). The LER values for grain legumes indicated that pea and chickpea achieved only about half of their biomass production (0.53 and 0.51, respectively), whereas lentil nearly reached its full potential (0.95). Cereal rye LER in intercrops with pea and lentil was greater than 1, although the interactions differed: pea likely competed with cereal rye, whereas lentil did not. In the WR+Ch intercrop, growing conditions, including potential allelopathic effects, were unfavorable for both crops. All intercrops exhibited LER total values greater than 1. The total LER ranged from 1.19 (WR+Ch) in the cereal rye–chickpea intercrop to 2.39 (WR+L) in the cereal rye–lentil intercrop (the differences were significant).

## 4. Discussion

The greenhouse assays were designed to isolate biochemical effects, whereas the field experiment reflects integrated ecological interactions. The limited correspondence between these results indicates that allelopathy operates within a broader context of resource competition and environmental modulation rather than as a dominant driver of crop interactions. Another limitation is that the greenhouse bioassays and field experiments involved different legume species. Crimson clover was used as a model species for preliminary screening of allelopathic activity under controlled conditions, whereas pea, lentil, and chickpea were evaluated under field conditions. In addition, radish was used as a standard sensitive indicator species for detecting phytotoxic effects during the preliminary bioassays and was not intended to simulate the response of the field-grown legume species. Consequently, the greenhouse results should be interpreted as evidence of potential allelopathic activity rather than as a direct representation of the field species combinations. Allelopathic activity was detectable under controlled conditions, as residue-derived leachates affected early radish root development; however, these effects were transient and not species-specific. Significant inhibition was observed only at 9 days after plant termination and only relative to the control, while no differences were detected among cereal rye, crimson clover, and substrate treatments. These findings suggest that the observed responses reflect short-term decomposition-related processes rather than distinct allelopathic dominance of individual species, supporting the concept that allelopathic interactions are strongly context-dependent under field conditions [27]. In addition, glyphosate was used only during the greenhouse plant termination phase prior to residue decomposition. Although the strongest biological responses were observed several days after termination and are therefore more likely associated with decomposition-derived compounds, a potential influence of glyphosate on leachate composition cannot be completely excluded.

Secondary metabolite analysis revealed pronounced interspecific differentiation, particularly in belowground biomass (Table 2), suggesting differences in biochemical potential within the rhizosphere. However, the extract-based bioassays demonstrated a clear species- and concentration-dependent response pattern (Figure 2), with stimulatory effects at lower concentrations and inhibitory effects at higher concentrations, consistent with hormetic responses described for phenolic compounds [15]. This indicates that potential biological activity in the soil water solution depends not only on total phenolic content but also on compound composition, concentration, transformation processes, and recipient sensitivity [13,18]. Environmental modulation of phytotoxic activity in soil further depends on physicochemical and microbial factors [28], which may attenuate or transform allelochemical effects under field conditions. Recent reviews also highlight that cereal allelopathy encompasses diverse mechanisms with the potential to function like bioherbicides against susceptible weed species [29] and that allelochemical profiles of cereals vary in their activity against both monocot and dicot weeds [30]. Our insights reinforce the understanding that allelopathic outcomes are strongly dependent on biological context and specific donor–recipient interactions.

According to the literature, cereal rye allelopathic effects are considered strongest during the first 4–8 weeks after termination, corresponding to the critical establishment period of the main crop [19]. In this study, aboveground biomass accumulation was therefore evaluated at 5 and 8 weeks (Table 3), coinciding with this relatively high potentially active window. If allelopathy had been the dominant mechanism shaping interactions, a sustained reduction in legume biomass and yield reduction would be expected in intercrops. However, no significant differences were detected after 5 weeks, and although some biomass reductions were observed after 8 weeks, overall productivity patterns suggest that direct biochemical suppression was not the sole factor influencing crop interactions under field conditions. Such complementarity effects are widely recognized as key drivers of intercropping advantages [3,31].

This interpretation is supported by land equivalent ratio (LER) values (Table 4), which exceeded 1.0 in all intercrop treatments, particularly in the cereal rye–lentil combination. These results are consistent with previous studies reporting positive interactions and improved performance of intercrops compared with monocrops [32] and align with broader evidence linking crop diversification to enhanced system resilience and resource-use efficiency [33]. However, the specific mechanisms underlying these responses were not directly assessed in the present study. A further limitation of the present study is that field observations were conducted during a single growing season at one experimental location. Therefore, caution is required when extrapolating the observed responses to other environmental conditions, years, and production systems. Further multi-year and multi-location studies are needed to confirm the consistency of the reported species interactions. The comparatively lower performance of the cereal rye–chickpea intercrop may reflect differences in species compatibility or other ecological interactions, although the specific mechanisms responsible were not directly assessed. An alternative explanation for the comparatively poorer performance of the cereal rye–chickpea intercrop may be related to the biochemical characteristics of chickpea itself. Among the tested species, chickpea exhibited the highest concentration of belowground polyphenolic acids (Table 2), whereas the WR+Ch treatment resulted in the lowest cereal rye biomass among the intercrops (Table 3). Although the present study does not allow causal relationships to be established, these observations suggest that interactions between cereal rye and chickpea may not have been unidirectional and that chickpea-derived compounds could also have contributed to the observed responses.

Effective weed suppression by rye residues in field conditions depends not only on biochemical properties but also on biomass quantity and mulch structure. Mechanical suppression alone typically requires at least 9000 kg ha^−1^ of rye dry biomass [34], indicating that allelopathic impact is closely linked to residue density and environmental context. Moreover, living cereal rye roots are known to release benzoxazinoids and phenolic compounds into the rhizosphere, potentially affecting the germination of sensitive species. Sensitivity to residue-mediated suppression has been shown to vary with seed mass and early resource buffering capacity [35]. Large-seeded crops, including many grain legumes, may possess detoxification mechanisms that mitigate such effects [19], which could explain the absence of strong negative impacts on legume productivity in the present study.

Overall, the findings indicate that allelopathic processes in cereal rye–legume systems are detectable during early developmental stages and under controlled conditions, but under field conditions their expression is diluted within broader ecological interactions. The present results suggest that allelopathic effects may operate over relatively short temporal windows; however, their expression under field conditions is likely influenced by multiple interacting factors, including spatial crop arrangement, root system architecture, soil water dynamics, and soil microbial processes, as reported in previous studies [13,15]. Because these factors were not directly measured in the present study, their specific contribution to the observed responses cannot be determined. The absence of significant reductions in grain legume biomass does not preclude a potentially selective effect on more sensitive species, such as weeds [36], although this requires direct validation at the weed community level. Given the variability of weed suppression reported in intercropping systems involving allelopathic species, further research should focus on quantifying these selective effects under field conditions and improving our understanding of the mechanisms underlying weed suppression in diversified cropping systems. Such studies would help to optimize the functional integration of potentially allelopathic species within sustainable intercropping systems.

## 5. Conclusions

This study identified measurable biological activity consistent with allelopathic effects under controlled conditions; however, the specific mechanisms underlying crop interactions in the field were not directly assessed.

Land equivalent ratio (LER) values exceeding unity in all intercrops indicate more efficient resource use compared with monocropping, particularly in the cereal rye–lentil combination. These findings suggest that resource partitioning, spatial arrangement, and species compatibility may have contributed substantially to intercrop performance under field conditions, whereas direct allelopathic interference did not appear to be a dominant factor.

Overall, the results indicate that cereal rye has potential for integration as a living mulch in relay intercropping systems with grain legumes. However, intercrop performance varied among legume species, with the greatest advantage observed in the cereal rye–lentil combination. Further studies are needed to confirm the consistency of these findings under different environmental conditions.

## Figures and Tables

**Table 1:** Radish (*Raphanus sativus* L.) root length (mm) after incubation with aqueous leachates from cereal rye and crimson clover residues under greenhouse conditions.

Treatments	3 DAP			9 DAP			15 DAP		
	12 h	24 h	48 h	12 h	24 h	48 h	12 h	24 h	48 h
Control	11.51 a	47.5 a	73.3 a	7.88 a	68.18 a	110.6 a	9.34 a	36.1 a	69.3 a
Substrate	10.2 a	44.0 a	66.0 a	8.16 a	51.9 b	78.8 b	9.71 a	33.1 a	55.1 a
Clover	10.7 a	36.6 a	52.6 a	8.33 a	50.5 b	72.1 b	10.5 a	25.3 a	48.8 a
Cereal rye	11.8 a	46.5 a	72.1 a	8.70 a	49.4 b	70.7 b	9.50 a	33.2 a	50.6 a
Mean	10.96	42.86	64.54	8.35	52.37	77.52	9.85	31.10	53.28
SD	0.75	4.89	9.47	0.34	8.84	18.74	0.51	4.63	9.27
SE	0.37	2.45	4.74	0.17	4.42	9.37	0.26	2.31	4.63
R^2^	0.182	0.104	0.130	0.055	0.354	0.454	0.136	0.089	0.123
Pr > F	0.149	0.407	0.297	0.680	0.009	0.001	0.274	0.479	0.324

Note: Control—distilled water; Substrate—leachate from substrate without plants; Clover—leachate from crimson clover (*Trifolium incarnatum* L.) residues; cereal rye—leachate from cereal rye (*Secale cereale* L.) residues. DAP—days after plant termination (glyphosate application). SD—standard deviation; SE—standard error. Means followed by the same letters in the same column and DAP section do not differ significantly (*p* < 0.05).

**Table 2:** Aboveground and belowground biomass total content of polyphenols, flavonoids and polyphenolic acids.

	Aboveground Biomass			Underground Biomass		
Treatment	Total content of polyphenols, mg RUE */g	Total content of flavonoids, mg RUE/g	Total content of polyphenolic acids, mg CAE **/g	Total content of polyphenols, mg RUE */g	Total content of flavonoids, mg RUE/g	Total content of polyphenolic acids, mg CAE **/g
Cereal rye	17.23 ± 6.54 b	6.85 ± 1.99 a	16.82 ± 3.44 a	5.24 ± 1.43 c	1.81 ± 0.54 b	3.28 ± 0.73 c
Pea	30.69 ± 8.03 ba	20.84 ± 6.57 a	18.73 ± 6.23 a	19.51 ± 12.11 b	9.37 ± 5.16 a	22.48 ± 14.71 b
Lentil	16.88 ± 13.92 b	12.66 ± 10.11 ba	12.02 ± 8.92 a	9.61 ± 0.00 c	4.16 ± 0.00 b	19.65 ± 0.00 b
Chickpea	45.91 ± 8.29 a	17.7 ± 3.18 ba	19.59 ± 3.58 a	43.06 ± 3.03 a	8.83 ± 0.70 a	89.99 ± 4.14 a
SD	13.75	6.12	3.39	16.89	3.66	38.37
SE	6.88	3.06	1.69	8.45	1.83	19.19
Mean	27.68	14.51	16.79	19.35	6.04	33.85
R^2^	0.69709	0.51364	0.26518	0.92963	0.74701	0.97444
Pr > F	0.0180	0.1074	0.4561	<0.0001	0.0090	<0.0001

Note: SD—standard deviation, SE—standard error. RUE *—routine equivalent; CAE **—coffee acid equivalent. Means followed by the same letters in the same column and section do not differ from one another (*p* < 0.05).

**Table 3:** Aboveground biomass of cereal rye, grain legume monocrops and their intercrops at 5 and 8 weeks after emergence (values represent mean ± SD).

Treatment	Biomass of Cereal Rye kg ha^−1^ DM	Biomass of Legumes kg ha^−1^ DM	Total Biomass of Intercropped Legumes kg ha^−1^ DM	vs. Cereal rye Monocrop kg ha^−1^ DM *	vs. Legumes Monocrop kg ha^−1^ DM *	vs. 5 Weeks	
						Cereal Rye kg ha^−1^ DM **	Legumes kg ha^−1^ DM **
	After 5 weeks						
WR	1834.4 ±174.0 a		1834.4 ± 174.0 b				
P		4203.6 ±1225.5 a	4203.6 ± 1225.5 a				
L		3112.2 ±1311.5 a	3112.2 ± 1311.5 b				
Ch		4208.8 ±1615.9 a	4208.8 ± 1615.9 a				
WR+P	2012.6 ±353.7 a	3075.2 ±1486.6 a	5087.8 ± 1779.1 a	+178.2 ±459.4 a	−1128.4 ±1071.2 a		
WR+L	1996.8 ±149.6 a	2965.6 ±373.2 a	4962.4 ± 392.7 a	+162.4 ±121.4 a	−146.6 ±1243.3 a		
WR+Ch	1671.0 ±232.9 a	2849.8 ±420.6 a	4520.8 ± 321.9 a	−163.4 ±299.7 a	−1359.0 ±1413.3 a		
SD	138.7	574.4	1065.2	157.4	525.7		
SE	160.2	629.2	1150.6	192.8	643.8		
Mean	1878.7	3402.5	3990.0	59.1	−878.0		
R-Square	0.2491	0.1915	0.4610	0.1907	0.1502		
Pr > F	0.3113	0.5309	0.0284	0.3860	0.4808		
	After 8 weeks						
WR	2545.0 ±294.7 bc		2545.0 ± 294.7 d			+710.6 ± 369.3 ba	
P		5776.2 ±1936.5 b	5776.2 ±1936.5 bc				+1572.6 ±896.7 a
L		4232.8 ±852.9 b	4232.8 ±852.9 dc				+1120.6 ±576.9 b
Ch		9241.8 ±221.6 a	9241.8 ±221.6 a				+5033.0 ±741.4 b
WR+P	3081.2 ±765.7 ab	3363.0 ±1826.3 b	6444.2 ±1647.7 bac	+536.2 ±918.9 ab	−2413.2 ±314.3 b	+1068.6 ±793.6 a	+287.8 ±628.1 b
WR+L	3558.2 ±624.7 a	4116.0 ±1524.6 b	7674.,2 ±1737.5 ab	+1013.2 ±917.6 a	−116.8 ±898.4 a	+1561.4 ±539.5 a	+1003.8 ±701.4 b
WR+Ch	1713.4 ±161.4 c	4633.0 ±1643.1 b	6346.4 ±1695.8 abc	−831.6 ±242.1 b	−4608.8 ±1864.1 b	+42.4 ±367.4 b	+1783.2 ±1376.5 b
SD	685.0	1935.2	2025.9	781.9	1834.0	553.5	1521.3
SE	791.0	2119.9	2188.2	957.6	1005.0	639.1	1666.5
Mean	2724.4	5227.1	6037.2	239.3	−2379.6	845.8	1800.2
R-Square	0.6327	0.5587	0.6137	0.5124	0.6973	0.5069	0.7566
Pr > F	0.0060	0.0073	0.0014	0.0395	0.0046	0.0320	<0.0001

Note. Monocrops: WR—cereal rye, P—pea, L—lentil, Ch—chickpea; relay-row intercrops: WR+P—rye intercropped with pea, WR+L—rye intercropped with lentil, WR+Ch—rye intercropped with chickpea; SD—standard deviation, SE—standard error. Means followed by the same letters in the same column and section do not differ from one another (*p* < 0.05). * Calculated difference between plant biomass grown in intercropping and monocropping systems. ** Calculated difference between biomass at 8 weeks and the corresponding treatment at 5 weeks.

**Table 4:** Total aboveground dry mass, species contribution and land equivalent ratio (LER) of cereal rye–grain legume monocrops and intercrops.

Treatment	After 5 Weeks	After 8 Weeks					
	Total Aboveground Biomass Kg ha^−1^ DM	Total Aboveground Biomass Kg ha^−1^ DM	Grain Legume Fraction, %	Cereal Rye Fraction, %	LER Grain Legume	LER Cereal Rye	LER Total
WR	1834.4 ± 174.0 b	2545.0 ± 294.7 a		100.0			
P	4203.6 ± 1225.5 a	5776.2 ± 1936.5 b	100.0				
L	3112.2 ± 1311.5 b	4232.8 ± 852.9 b	100.0				
Ch	4208.8 ± 1615.9 a	9241.8 ± 2216.6 b	100.0				
WR+P	5087.8 ± 1779.1 a	6444.2 ± 1647.7 ab	52.2	47.8	0.53 ± 0.19 b	1.24 ± 0.38 ab	1.77 ± 0.38 ab
WR+L	4962.4 ± 392.7 a	7674.2 ± 1737.5 ab	53.6	46.4	0.95 ± 0.23 a	1.44 ± 0.40 a	2.39 ± 0.46 a
WR+Ch	4520.8 ± 321.9 a	6346.0 ± 1695.8 ab	73.0	27.0	0.51 ± 0.16 b	0.68 ± 0.07 b	1.19 ± 0.19 b
SD	1065.2	2025.9			0.20	0.32	0.49
SE	1150.6	2188.2			0.25	0.40	0.60
Mean	3990.0	6037.2			0.66	1.12	1.78
R^2^	0.4610	0.614			0.5215	0.5015	0.6494
Pr > F	0.0284	0.001			0.0363	0.0436	0.0089

Note. Monocrops: WR—cereal rye, P—pea, L—lentil, Ch—chickpea; relay-row intercrops: WR+P—rye intercropped with pea, WR+L—rye intercropped with lentil, WR+Ch—rye intercropped with chickpea; SD—standard deviation, SE—standard error. Means followed by the same letters in the same column and section do not differ from one another (*p* < 0.05).

## Data Availability

All data generated or analyzed during this study are included in this published article.
